# Childhood Community Disadvantage and MRI-Derived Structural Brain Integrity After Age 65 Years

**DOI:** 10.1001/jamanetworkopen.2024.43703

**Published:** 2024-11-07

**Authors:** Rachel L. Peterson, Erika Meza, Kristen M. George, Pauline Maillard, Charles DeCarli, Paola Gilsanz, Yenee Soh, Yi Lor, Amy J. Kind, Lisa L. Barnes, Rachel A. Whitmer

**Affiliations:** 1 University of Montana, Missoula; 2 Harvard T.H. Chan School of Public Health, Boston, Massachusetts; 3 University of California, Davis; 4 Kaiser Permanente Northern California, Oakland; 5 University of Wisconsin, Madison; 6 Rush University, Chicago, Illinois

## Abstract

**Importance:**

Prior studies associate late-life community disadvantage with worse brain health. It is relatively unknown if childhood community disadvantage associates with late-life brain health.

**Objective:**

To test associations between childhood residence in an economically disadvantaged community, individual income and education, and late-life cortical brain volumes and white matter integrity.

**Design, Setting, and Participants:**

This cohort study was conducted in the ongoing harmonized cohorts KHANDLE (Kaiser Healthy Aging and Diverse Life Experiences Study; initiated 2017) and STAR (Study of Healthy Aging in African Americans; initiated 2018) using all available data collected out of a regional integrated health care delivery network in California between cohort initiation and analysis initiation in June 2023. Eligible participants were Kaiser Permanente Northern California member ages 65 years or older. Data were analyzed between June and November 2023.

**Exposure:**

Residence at birth was geocoded and linked to historical Area Deprivation Indices (ADI). ADI is a nationally ranked percentile; community disadvantage was defined as ADI of 80 or higher.

**Main Outcomes and Measures:**

Regional brain volumes and white matter integrity measures were derived from a random subset of participants who underwent 3T magnetic resonance imaging. Models adjusted for race and ethnicity, sex, and parental education.

**Results:**

Of a total 2161 individuals in the combined cohort, 443 individuals were eligible for imaging (mean [SD] age, 76.3 [6.5] years; 253 female [57.1%]; 56 Asian [12.6%], 212 Black [47.9%], 67 Latino [15.1%], 109 White [24.6%]). Imaging participants had a mean (SD) 15.0 (2.5) years of education, and 183 (41.3%) earned $55 000 to $99 999 annually. Fifty-four participants (12.2%) resided in a disadvantaged childhood community. Childhood community disadvantage was associated with smaller gray matter volumes overall (−0.39 cm^3^; 95% CI, −0.65 to −0.10 cm^3^) and in the cerebellum (−0.39 cm^3^; 95% CI, −0.66 to −0.09 cm^3^), hippocampus (−0.37 cm^3^; 95% CI, −0.68 to −0.04 cm^3^), and parietal cortex (−0.25 cm^3^; 95% CI, −0.46 to −0.04 cm^3^) and larger mean lateral ventricle (0.44 cm^3^; 95% CI, 0.12 to 0.74 cm^3^), third ventricle (0.28 cm^3^; 95% CI, 0.03 to 0.55 cm^3^), and white matter hyperintensity volume (0.31 cm^3^; 95% CI, 0.06 to 0.56 cm^3^). Educational attainment and late-life income did not mediate these associations.

**Conclusions and Relevance:**

In this cohort study of racially and ethnically diverse health plan members, childhood community disadvantage was associated with worse late-life brain health independent of individual socioeconomic status. Future work should explore alternative pathways (eg, cardiovascular health) that may explain observed associations.

## Key Points

**Question:**

Is childhood community disadvantage associated with magnetic resonance imaging–derived late-life brain volumes, independent of individual socioeconomic status?

**Findings:**

In this cohort study of 443 adults ages 65 years or older from diverse racial and ethnic populations, residing in a disadvantaged community in childhood was significantly associated with smaller gray matter in the cerebellum, hippocampus, and parietal cortex and with larger lateral ventricle volume and greater white matter hyperintensity volume.

**Meaning:**

These results suggest that historical economic residential segregation experienced in childhood has lasting associations with structural brain volumes not explained by individual income or educational attainment.

## Teaser

This cohort study of US adults in a regional integrated health care network examines the association of childhood residence in an economically disadvantaged community, individual income and education, and late-life cortical brain volumes and white matter integrity.

## Introduction

Community socioeconomic disadvantage is a well-established social determinant of health and key component of the social exposome—the totality of the social environment exposures at individual, interpersonal, and societal levels experienced across the lifecourse.^1,2^ Recent studies associate community disadvantage in late life with reduced hippocampal and other regional brain volumes,^3^ cortical thinning,^4^ higher dementia incidence,^5,6^ and increased odds of having Alzheimer disease neuropathology at death.^7^ In children and adolescents, community disadvantage has been associated with smaller regional brain volumes,^8,9^ cortical thinning,^10,11^ and greater amygdala reactivity^12,13^ independent of family socioeconomic status (SES). These studies, which assess community disadvantage within a few years of neuroimaging assessment, are important contributions to understanding linkages between the social exposome and brain health. However, we are not aware of any studies that have examined the associations between residing in a socioeconomically disadvantaged community in childhood and structural brain integrity decades later in late life. Because early childhood is a sensitive period for neurological development, examining associated outcomes over the lifecourse may help to inform interventions targeting the social exposome as a modifiable risk factor for dementia.

This research is especially important in minoritized racial or ethnic groups for which dementia disparities have been documented.^14^ Residence in disadvantaged communities is not random but driven by historical and contemporary systems that concentrate low-income and minoritized populations in communities with fewer resources and more health-harming exposures.^15,16^ Yet, most research examining associations of community disadvantage and late-life brain health have been conducted in predominately White populations, limiting inference to the groups overrepresented in disadvantaged communities. Expanding research to racially and ethnically diverse groups may better inform intervention targets that also reduce dementia disparities.

Studies of community-based SES require appropriate methods to accurately account for variation in individual SES (eg, individual educational attainment and household income). Prior work has shown that individual SES across the lifecourse is an important predictor of late-life cognitive and brain health.^17,18,19,20,21^ The individual or familial resources made available via education and income, such as better nutrition or lower stress, may mediate associations of community disadvantage and brain health outcomes.^22,23,24^

We aimed to address these gaps. Using data from a racially and ethnically diverse cohort, we estimated associations between childhood community-level disadvantage and late-life gray matter cortical thinning, ventricle volumes, and white matter integrity as well as the extent to which observed associations are explained (ie, mediated) by individual educational attainment and late-life income.

## Methods

The Kaiser Healthy Aging and Diverse Life Experiences (KHANDLE) Study and the Study of Healthy Aging in African Americans (STAR) are harmonized longitudinal cohorts of long-term Kaiser Permanente Northern California members living in the San Francisco Bay area and Sacramento Valley. Participants were eligible for KHANDLE if they were age 65 years or older on January 1, 2017, spoke English or Spanish, and participated in voluntary multiphasic health check-ups (MHC) from 1964 to 1985. Stratified random sampling by race, ethnicity, and educational attainment was used to recruit approximately equal proportions of Asian, Black, Latino, and White participants. Participants eligible for STAR self-identified as Black or African American, were age 50 years or older on January 1, 2018, participated in MHC, and spoke English or Spanish. Stratified random sampling by age and educational attainment was used to recruit approximately equal proportions of Black participants ages 50 to 64 years and 65 years and older. Exclusion criteria for both studies included electronic medical record diagnosis of dementia or another neurodegenerative disease (frontotemporal dementia, Lewy body disease, Pick disease, Parkinson disease with dementia, Huntington disease) and the presence of health conditions that would impede participation in study interviews (defined by hospice activity in the past 12 months, history of severe chronic obstructive pulmonary disease in the past 6 months, congestive heart failure hospitalizations in the past 6 months, and history of end-stage kidney disease or dialysis in the past 12 months) at time of enrollment.

A stratified random sample of 25% of KHANDLE and STAR participants was selected for brain imaging using 3T Siemens Tim Trio magnetic resonance imaging (MRI) housed in the Imaging Research Center of University of California Davis Medical Center. This study used imaging data collected and prepared for analyses between study onset and June 2023 and followed the Strengthening the Reporting of Observational Studies in Epidemiology (STROBE) reporting guideline for cohort studies. KHANDLE and STAR were institutional review board approved by Kaiser Permanente Northern California and the University of California, Davis. All participants provided written informed consent.

### Measures

MRI-derived outcome measures included atlas-based regional cortical brain volumes, hippocampal volume, and lobar white matter lesion volumes.^25,26,27,28^ DTI-based measures of free water and fractional anisotropy, sensitive indicators of white matter integrity, were assessed using algorithms described elsewhere.^25,26^ These measures were selected for this current study as they are common measures of brain aging and injury associated with age-related neurodegeneration. Detailed methods for imaging and deriving MRI measures is provided in the eAppendix in Supplement 1. Imaging measures for gray matter, white matter hyperintensity, and ventricular volumes were normalized by intracranial volume. White matter hyperintensity was log-transformed to account for a skewed distribution. All imaging measures were z-standardized.

Childhood community disadvantage was based on address at birth self-reported during the baseline interview. Addresses were validated and geocoded, then assigned a county-level Area Deprivation Index (ADI) based on the decade in which the participant resided at that address (1920 through 1970). ADIs were calculated and provided by the University of Wisconsin Center for Health Disparities Research, following previously described methods.^29,30^ Briefly, ADIs were derived from publicly available US decennial census data for each decade. Scores were scaled nationally by census decade such that an ADI of 1 represents the 1% of most advantaged geographic units in the US during that decade, and an ADI of 100 represents the least advantaged 1% of geographic units in the US during that same decade. An ADI for any geographic unit could vary decade to decade, reflecting the social exposome of the time.

If a geocoded address at birth was missing, we imputed ADI scores from addresses reported for age 5 years or age 10 years. Prior work in these cohorts and other datasets have shown that the health effects of neighborhood SES may be strongest at the most extreme levels.^31,32^ Given our sample is skewed toward having more advantaged childhood community contexts (eFigure 1 in Supplement 1), we operationalized community disadvantage as having a childhood ADI of 80 or higher vs an ADI below 80 (the reference category) to ensure adequate sample size while aligning with approaches used in other studies.

We evaluated education and late-life income as individual markers of SES and potential mediators. Years of completed education was derived from 2 variables. For participants with a high school diploma or higher, numerical values (12 to 20 years) were assigned to responses from a categorical variable of highest level of education completed (ie, high school diploma, some college, bachelor’s degree, master’s degree, doctoral degree). For participants with less than a high school diploma, years of completed schooling (zero to 11 years) were reported. Income reported categorically was recoded to be continuous based on the highest income in each categorical range (eg, $55 000 to $64 999 was numerically assigned as 64 999).

Covariates included parental educational attainment as a measure of childhood family SES. Maternal and paternal education was reported separately as (1) years completed if less than a high school diploma; or (2) highest degree attained. We calculated years of education for each parent separately with 12 indicative of a high school diploma and 16 of a bachelor’s degree. When parental education was missing, it was coded as zero and an indicator variable was included in adjusted analyses to differentiate those with zero years of parental education from those missing parental education to avoid bias resulting from complete case analysis when missingness is not at random.^33^ Additional covariates included sex (self-reported or, when missing, pulled from electronic medical records as female or male) and self-reported race or ethnicity was classified as Asian, Black, Latino, and White.

### Statistical Analysis

We restricted the combined KHANDLE and STAR neuroimaging subsample to participants ages 65 years and older who had a valid geocoded childhood address that mapped to area deprivation indices. Most participants missing geocoded addresses at all 3 childhood ages immigrated to the US after age 10 years. Participants also were excluded if missing responses on demographic covariates. Our final analytic sample was 443 participants (eFigure 2 in Supplement 1).

We compared demographic characteristics of the combined KHANDLE and STAR cohort ages 65 years and older with our imaging analytic sample. We utilized a causal mediation framework to estimate the average direct effects (ADE) of childhood community disadvantage on structural brain volumes and the average causal mediated effects (ACME) of participants’ education or late-life income on associations of childhood community disadvantage and structural brain volumes. Causal inference methods are a useful tool to examine treatments or exposures that cannot be ethically or practicably randomized. The causal mediation approach used in this study approximates randomization by holding treatment (ie, childhood community disadvantaged) constant while estimating the difference in structural brain volumes that corresponds with the expected years of educational attainment or household income, thereby isolating the mediator as the potential mechanism of the association. The key assumption in this approach is no unmeasured confounding of the exposure-mediator-outcome (pretreatment confounding) or mediator-outcome (posttreatment confounding) associations (eFigure 3 in Supplement 1).^34,35^

Specifically, we fit a series of 2 linear regression models for each mediator-outcome pairing. The outcome models estimated the association of childhood community disadvantage with each imaging marker of structural brain integrity as dependent variables, adjusting for the mediators, race and ethnicity (as a proxy for racism), sex, and parental education. Model parameters were simulated using 1000 draws from the distribution to estimate (1) the potential values of the mediator under the exposed condition and nonexposed condition and (2) 2 potential outcomes given the simulated values of each mediator under the exposed and nonexposed values. The mediator models estimated the association of childhood community disadvantage on participant educational attainment or income as the dependent variables, with adjustment for confounding by race and ethnicity, sex, and parental education. The ACME is calculated as the mean difference in outcome that would correspond with changing the mediator from the value it would take under the exposed condition and the value it would take under the control condition.^34^ Because both our outcome and mediator models employed linear regression, the ACME, or indirect effect, corresponds to the product of coefficients methods commonly used in traditional mediation analyses (*y = ab + c’*). Standard errors for all mediator and outcome regression models were bootstrapped with 500 replications. Analyses were undertaken from June to November 2023 and were conducted in Stata version 17.0 (StataCorp LLC). All mediation models were estimated in the Mediation package.^34^

## Results

Out of a total 2161 individuals in the combined KHANDLE and STAR cohorts, 443 were included in the final analytic sample (mean [SD] age, 76.3 [6.5] years; 253 female [57.1%]; 56 Asian [12.6%], 212 Black [47.9%], 67 Latino [15.1%], 109 White [24.6%]). Compared with KHANDLE and STAR cohorts of participants ages 65 years and older, participants in the analytic sample were slightly older (mean [SD] age, 76.3 [6.5] years vs 75.8 [7.1] years), had slightly more education (mean [SD], 15.0 [2.5] years vs 14.6 [3.0] years), and were less likely to be from a disadvantaged childhood community (54 of 443 [12.2%] vs 283 of 2161 [16.1%]) (Table 1). The analytic sample also was more likely to report maternal education as greater than high school (252 of 443 [56.9%] vs 1070 of 2161 [49.5%]) and less likely to earn less than $55 000 a year in late life (130 of 443 [29.3%] vs 712 of 2161 [32.9%]). Significant differences between the analytic sample and those missing a childhood address are lower prevalence of birth outside the US (15 of 500 [3.0%] vs 73 of 79 [92.4%]), younger age (mean [SD], 76.4 [6.5] years vs 78.5 [5.4] years) and slightly higher maternal education (mean [SD], 10.9 [4.0] years vs 9.5 [4.6] years). Measures of community-level and individual-level SES were weakly correlated, ranging from −0.26 for childhood ADI (where higher scores indicate less advantaged communities) and paternal education to −0.17 between childhood ADI and participant education (eTable in Supplement 1).

**Table 1.** Characteristics of Participants Aged 65 Years or Older in the Combined KHANDLE and STAR Imaging Sample Overall and by Childhood Community Advantage Assessed by the Area Deprivation Index

Characteristics	Individuals, No. (%)			
	Combined KHANDLE and STAR cohorts (n = 2161)	Analytic sample (n = 443)	Childhood community	
			More advantaged (high SES) (n = 389)	Disadvantaged (low SES) (n = 54)
Age, mean (SD), y	75.8 (7.1)	76.3 (6.5)	75.7 (6.1)	81.1 (7.3)
Sex				
Female	1309 (60.6)	253 (57.1)	217 (55.8)	36 (66.7)
Male	852 (39.4)	190 (42.9)	172 (44.2)	18 (33.3)
Education, mean (SD), y	14.6 (3.0)	15.0 (2.5)	15.1 (2.5)	14.0 (2.1)
Childhood community disadvantage	283 (16.1)^a^	54 (12.2)	0	54 (100)
Childhood ADI, mean (SD)	28.9 (34.4)^a^	23.8 (31.7)	14.5 (21.0)	90.5 (5.0)
Income				
Not reported	161 (7.5)	0	0	0
<$55 000	712 (32.9)	130 (29.3)	103 (26.5)	27 (50.0)
$55 000-$99 999	738 (34.2)	183 (41.3)	166 (42.7)	17 (31.5)
≥$100 000	550 (25.5)	130 (29.3)	120 (30.8)	10 (18.5)
Parental education				
Maternal education, mean (SD), y	10.3 (4.3)	10.9 (4.0)	11.1 (4.0)	9.4 (3.9)
Maternal education high school diploma or higher	1070 (49.5)	252 (56.9)	231 (59.4)	21 (38.9)
Maternal education missing	364 (16.8)	57 (12.9)	47 (12.1)	10 (18.5)
Paternal education, mean (SD), y	10.6 (4.3)	11.0 (4.7)	11.3 (4.6)	8.5 (5.0)
Paternal education high school diploma or higher	949 (43.9)	210 (47.4)	195 (50.1)	15 (27.8)
Paternal education missing	558 (25.8)	97 (21.9)	80 (20.6)	17 (31.5)
Race and ethnicity				
Asian	415 (19.2)	56 (12.6)	52 (13.4)	4 (7.4)
Black	895 (41.4)	212 (47.9)	178 (45.8)	34 (63.0)
Latino	349 (16.1)	67 (15.1)	59 (15.2)	8 (14.8)
White	502 (23.2)	109 (24.6)	100 (25.7)	8 (14.8)

Abbreviations: ADI, Area Deprivation Index; KHANDLE, Kaiser Healthy Aging and Diverse Life Experiences Study; STAR, Study of Healthy Aging in African Americans.

^a^ Based on a sample of 1761 participants with valid childhood addresses.

**Figure 1.**
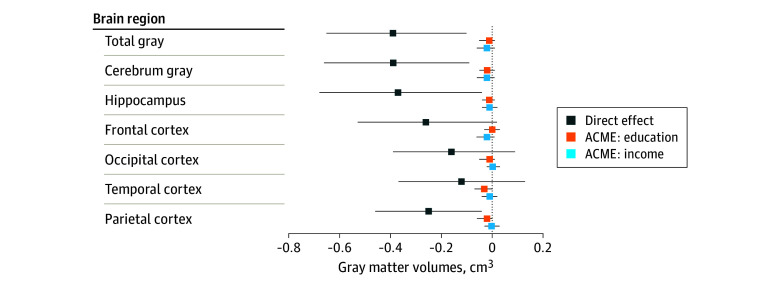
Estimated Average Direct Effects of Childhood Community Disadvantage on Late-Life Gray Matter Volumes and Average Causal Mediated Effects (ACME) of Education and Income on the Association of Childhood Community Disadvantage and Late-Life Gray Matter Volumes All models adjusted for sex, race and ethnicity, and parental educational attainment.

**Figure 2.**
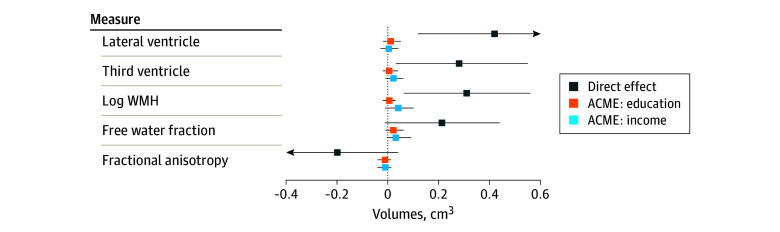
Estimated Average Direct Effects of Childhood Community Disadvantage on Late-Life Gray Matter Volumes and Average Causal Mediated Effects (ACME) of Education and Income on the Association of Childhood Community Disadvantage and Late-Life Ventricular Volumes and White Matter Integrity All models adjusted for sex, race and ethnicity, and parental educational attainment. WMH indicates white matter hyperintensity.

In our mediation analyses, the average direct effects (ADE) of childhood community disadvantage on gray matter volumes indicated smaller total volumes (−0.39 cm^3^; 95% CI, −0.65 to −0.10 cm^3^), and smaller cerebellum (−0.39 cm^3^; 95% CI, −0.66 to −0.09 cm^3^), hippocampus (−0.37 cm^3^; 95% CI, −0.68 to −0.04 cm^3^), and parietal cortex volumes (−0.25 cm^3^; 95% CI, −0.46 to −0.04 cm^3^) (Table 2, Figure 1). We observed nonsignificant differences in the frontal (−0.26 cm^3^; 95% CI, −0.53 to 0.02 cm^3^), occipital (−0.16 cm^3^; 95% CI, −0.39 to 0.09 cm^3^), and temporal (−0.12 cm^3^; 95% CI, −0.38 to 0.18 cm^3^) cortices. The ADE of childhood community disadvantage was also associated with significantly larger mean lateral ventricle volume (0.44 cm^3^; 95% CI, 0.12 to 0.74 cm^3^), and third ventricle volume (0.28 cm^3^; 95% CI, 0.03 to 0.55 cm^3^) (Table 2, Figure 2). ADE estimates also indicated associations of childhood community disadvantage and compromised white matter integrity; specifically, larger greater white matter hyperintensity volume (0.31 cm^3^; 95% CI, 0.06 to 0.56 cm^3^). Differences in free water fraction (0.21 cm^3^; 95% CI, −0.01 to 0.44 cm^3^) and fractional anisotropy (−0.20 cm^3^; 95% CI, −0.42 to 0.04 cm^3^) were not statistically significant.

**Table 2.** Average Direct Effect Estimates of Childhood Community Disadvantage and Late-Life Brain Health Markers and Average Causal Mediated Effects Through Participant Education and Income^a^^,^^b^

Measure	Gray matter volume, mean (bootstrapped 95% CI), cm^3^			
	Direct effect		Mediated via education	Mediated via income
Cerebrum (total volume)	−0.39 (−0.65 to −0.10)	−0.01 (−0.05 to 0.01)		−0.02 (−0.06 to 0.01)
Cerebellum	−0.39 (−0.66 to −0.09)	−0.02 (−0.05 to 0.01)		−0.02 (−0.06 to 0.01)
Hippocampus	−0.37 (−0.68 to −0.04)	−0.01 (−0.04 to 0.01)		−0.01 (−0.04 to 0.02)
Frontal cortex	−0.26 (−0.53 to 0.02)	0.001 (−0.03 to 0.03)		−0.02 (−0.06 to 0.01)
Occipital cortex	−0.16 (−0.39 to 0.09)	−0.01 (−0.05 to 0.01)		0.002 (−0.02 to 0.03)
Temporal cortex	−0.12 (−0.38 to 0.18)	−0.03 (−0.07 to 0.01)		−0.01 (−0.04 to 0.02)
Parietal cortex	−0.25 (−0.46 to −0.04)	−0.02 (−0.06 to 0.001)		−0.002 (−0.03 to 0.03)
Cerebrospinal fluid volumes				
Lateral ventricle	0.44 (0.12 to 0.74)	0.01 (−0.02 to 0.05)		0.002 (−0.03 to 0.04)
Third ventricle	0.28 (0.03 to 0.55)	0.003 (−0.02 to 0.04)		0.02 (−0.01 to 0.06)
Measures of white matter integrity				
Log white matter hyperintensities	0.31 (0.06 to 0.56)	0.004 (−0.02 to 0.03)		0.04 (−0.01 to 0.10)
Free water fraction, cm^3^	0.21 (−0.01 to 0.44)	0.02 (−0.01 to 0.06)		−0.03 (−0.004 to 0.09)
Fractional anisotropy	−0.20 (−0.42 to 0.04)	−0.01 (−0.04 to 0.01)		−0.01 (−0.04 to 0.01)

^a^ All models adjust for sex, race and ethnicity, and maternal and paternal education.

^b^ Average causal mediated effect is calculated as the mean difference in brain volume for each region that would correspond with changing the mediator from the value it would take under the exposed condition (disadvantaged childhood community) and the value it would take under the control condition (nondisadvantaged childhood community).

The direct effects of childhood community disadvantage on education and late-life income were nonsignificant but suggested an average of approximately half a year less education (−0.48 years; 95% CI, −1.17 to 0.21 years) and nearly $10 000 less in annual late-life income (−$9700; 95% CI, −$22 029 to $2629). The average causal mediated effects (ACME) of both educational attainment and late-life income were close to the null for all models (Table 2, Figures 1 and 2).

## Discussion

In our racially and ethnically diverse cohort of adults ages 65 years and older, we observed a pattern whereby residing in a disadvantaged community in childhood was associated with worse late-life brain health, as assessed by structural MRI-derived gray matter and ventricular volumes and white matter integrity. These models, adjusted for parental educational attainment as a marker of childhood SES, estimated that individual educational attainment and late-life income as markers of adulthood SES had limited explanatory effect on these associations. Put simply, our findings suggest that the association of living in a disadvantaged community in childhood and late-life structural brain health is independent of childhood familial and adulthood individual SES.

Our work builds upon a growing body of research that associates community disadvantage with reduced brain tissue volumes and cortical thinning among both older adults^3,4^ and children,^8,9,10,11^ independent of individual or family SES. However, prior work has relied on community disadvantage exposures that were concurrent or recent to neuroimaging assessment. We make an important contribution to social exposome research by expanding the time between childhood community disadvantage exposure and brain health assessment by more than 50 years.

Our findings add to a growing body of research highlighting the importance of the social exposome in childhood for dementia risk. Prior work has shown that various childhood social contexts, including residing in a household with few books,^36^ in rural areas,^37,38^ or in economically disadvantaged communities,^32^ are associated with worse late-life cognition. Other studies have found that birth in what has been described as a “stroke belt” state—a region of the southeast US with high stroke mortality—is associated with higher dementia incidence and poorer late-life cognition, even among those who moved away from the region.^39,40,41,42,43^ Given that modifiable risks for Alzheimer disease and related dementias (ADRD) accumulate across the lifecourse, community disadvantage may be a primordial cause of ADRD, contributing to the presence of risk factors that occur later in the lifecourse.^44^

Although we did not find evidence to suggest that individual SES explains the examined associations, several alternative potential pathways may link the childhood social exposome with later-life brain health. One possibility is exposure to a higher concentration of environmental contaminants, which is more common in disadvantaged communities. Air pollution is recognized as a key modifiable risk factor for dementia,^44^ and recent studies have found that children exposed to higher concentrations air pollution have smaller gray matter and cortical volumes when compared to nonexposed children.^45,46^ Alternatively, higher exposure to stressful and traumatic experiences, such as higher crime rates, may also help explain our findings, as suggested by studies examining childhood adversity and structural brain volumes.^47^

More broadly, this study contributes to evidence suggesting more attention should be paid to the early life social exposome. Increasing racial and economic segregation has unequally distributed harmful contextual exposures to minoritized and low-income populations.^48,49^ Public health interventions that target the early life social exposome are already under way in some communities, reducing the inequitable exposure to health-harming risk factors.^50^

### Strengths and Limitations

This study had several strengths. These include the availability of geocoded residential histories from childhood, measures for both childhood and late-life individual socioeconomic status, and a racially and ethnically heterogenous sample with neuroimaging. Future research will continue to explore potential mechanistic pathways by which childhood community disadvantage may contribute to late-life brain health, including modifiable health behaviors and cardiovascular risk factors.

We note several limitations. Causal mediation models require strong assumptions such that there is no unmeasured confounding in the exposure-outcome association, the exposure-mediator association, or the mediator-outcome association.^35^ Although we carefully selected confounders, we cannot rule out the possibility of unmeasured confounding. While parental education is a strong indicator of childhood familial SES, unmeasured aspects of familial SES may still confound the association. A second limitation is quantifying a dose-response association between duration of neighborhood exposure and late-life brain health, as our analysis relies on the ADI from the first childhood address available and does not account for how many years the child lived at this address. Nonetheless, because childhood ADI is derived at the county level, any relocations would need to be geographically distanced and to communities with drastically different socioeconomic status to bias results—a phenomena we expect is uncommon. While a major strength of this study is the racial and ethnic heterogeneity of the cohort, we are underpowered to examine patterns in our models that are specific to race or ethnicity. Future work should explore if the associations between community disadvantage on brain health are modified by racialized experiences via assessment of discrimination experiences or using race or ethnicity as a proxy. Additionally, MRI was only completed for a subsample of the KHANDLE and STAR cohorts. Although prior work has shown that Kaiser Permanente members generally are representative of the state populations where they reside,^51^ selection into the cohort and the MRI subsample may reduce generalizability. Lastly, because MRI was only completed once, we are unable to assess the onset of structural changes, or if childhood community disadvantage is associated with the rate of change. Repeat MRI is now under way in the KHANDLE cohort and future analyses will explore this question.

## Conclusions

In this cohort study, participants with high early life disadvantage had smaller gray matter volumes, larger ventricular volumes, and greater white matter hyperintensity volume in later life than participants without early life disadvantage. Expanding public health interventions targeting the early life social exposome may further prevent the emergence of risk factors for poor late-life brain health and reduce dementia disparities in the next generation.

## Notes

Supplement 1. **eTable.** Correlation Matrix of Community and Individual Level Markers of Socioeconomic Status in the KHANDLE and STAR Cohort Imaging Subsamples **eFigure 1.** Distribution of Childhood Area Deprivation Index Scores in the Analytic Sample of KHANDLE and STAR **eFigure 2.** Flow Diagram of the Inclusion and Exclusion Criteria for the Analytic Sample of the Kaiser Healthy Aging and Diverse Life Experiences (KHANDLE) and Study of Aging in African Americans (STAR) Cohorts **eFigure 3.** Directed Acyclic Graph of Tested Average Direct Effects (ADE) of Childhood Community Disadvantage on Structural Brain Volumes and Average Causal Mediated Effects (ACME) of Participants’ Education or Late-Life Income on Associations of Childhood Community Disadvantage and Structural Brain Volumes **eAppendix.** MRI Methods for the KHANDLE/STAR Cohorts

Supplement 2.
Data Sharing Statement
