# Supplementary material for: Childhood Community Disadvantage and MRI-Derived Structural Brain Integrity After Age 65 Years
Source: JAMA Netw Open. 2024 Nov 7;7(11):e2443703. doi: 10.1001/jamanetworkopen.2024.43703 (PMC11544493; doi:10.1001/jamanetworkopen.2024.43703)
Supplement: Supplement 1. — eTable. Correlation Matrix of Community and Individual Level Markers of Socioeconomic Status in the KHANDLE and STAR Cohort Imaging Subsamples eFigure 1. Distribution of Childhood Area Deprivation Index Scores in the Analytic Sample of KHANDLE and STAR eFigure 2. Flow Diagram of the Inclusion and Exclusion Criteria for the Analytic Sample of the Kaiser Healthy Aging and Diverse Life Experiences (KHANDLE) and Study of Aging in African Americans (STAR) Cohorts eFigure 3. Directed Acyclic Graph of Tested Average Direct Effects (ADE) of Childhood Community Disadvantage on Structural Brain Volumes and Average Causal Mediated Effects (ACME) of Participants’ Education or Late-Life Income on Associations of Childhood Community Disadvantage and Structural Brain Volumes eAppendix. MRI Methods for the KHANDLE/STAR Cohorts [file jamanetwopen-e2443703-s001.pdf]

Supplemental Online Content

Peterson RL, Meza E, George KM, et al. Childhood community disadvantage and MRI-Derived Structural Brain Integrity After Age 65 Years. *JAMA Netw Open.* 2024;7(11):e2443703. doi:10.1001/jamanetworkopen.2024.43703

eTable. Correlation Matrix of Community and Individual Level Markers of Socioeconomic Status in the KHANDLE and STAR Cohort Imaging Subsamples

eFigure 1. Distribution of Childhood Area Deprivation Index Scores in the Analytic Sample of KHANDLE and STAR

eFigure 2. Flow Diagram of the Inclusion and Exclusion Criteria for the Analytic Sample of the Kaiser Healthy Aging and Diverse Life Experiences (KHANDLE) and Study of Aging in African Americans (STAR) Cohorts

Figure 3. Directed Acyclic Graph of Tested Average Direct Effects (ADE) of Childhood Community Disadvantage on Structural Brain Volumes and Average Causal Mediated Effects (ACME) of Participants' Education or Late-Life Income on Associations of Childhood Community Disadvantage and Structural Brain Volumes

eAppendix. MRI Methods for the KHANDLE/STAR Cohorts

This supplemental material has been provided by the authors to give readers additional information about their work.

© 2024 Peterson RL et al. *JAMA Network Open.*

eTable. Correlation matrix of community and individual level markers of socioeconomic status in the KHANDLE and STAR cohort imaging subsamples

	Childhood ADI	Participant Education	Income	Maternal Education	Paternal Education
Childhood ADI	1				
Participant Education	-0.1697*	1			
Income	-0.1742*	0.3297*	1		
Maternal Education	-0.1847*	0.2966*	0.1696*	1	
Paternal Education	-0.2594*	0.2887*	0.2302*	0.5806*	1

*p<0.05

© 2024 Peterson RL et al. *JAMA Network Open.*

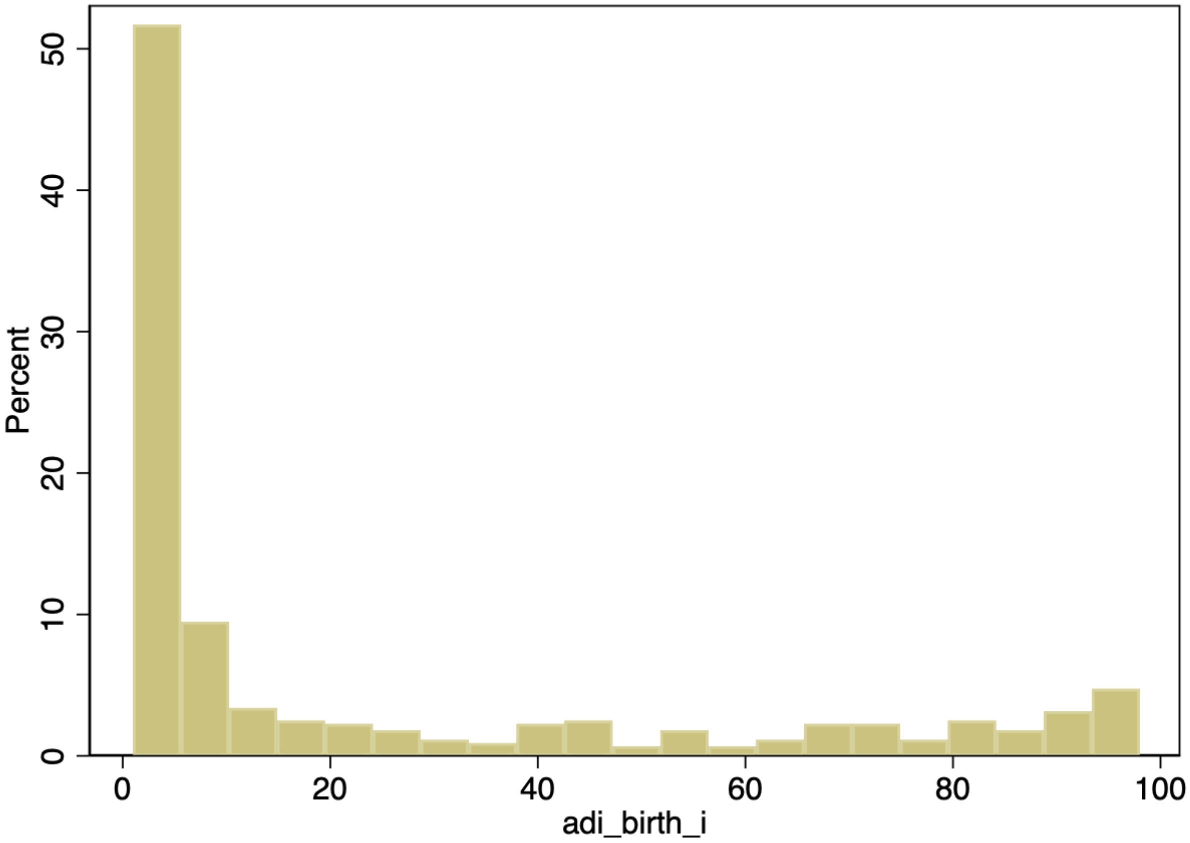

eFigure 1. Distribution of childhood area deprivation index scores in the analytic sample of KHANDLE and STAR. Higher values represent greater disadvantage.

© 2024 Peterson RL et al. *JAMA Network Open.*

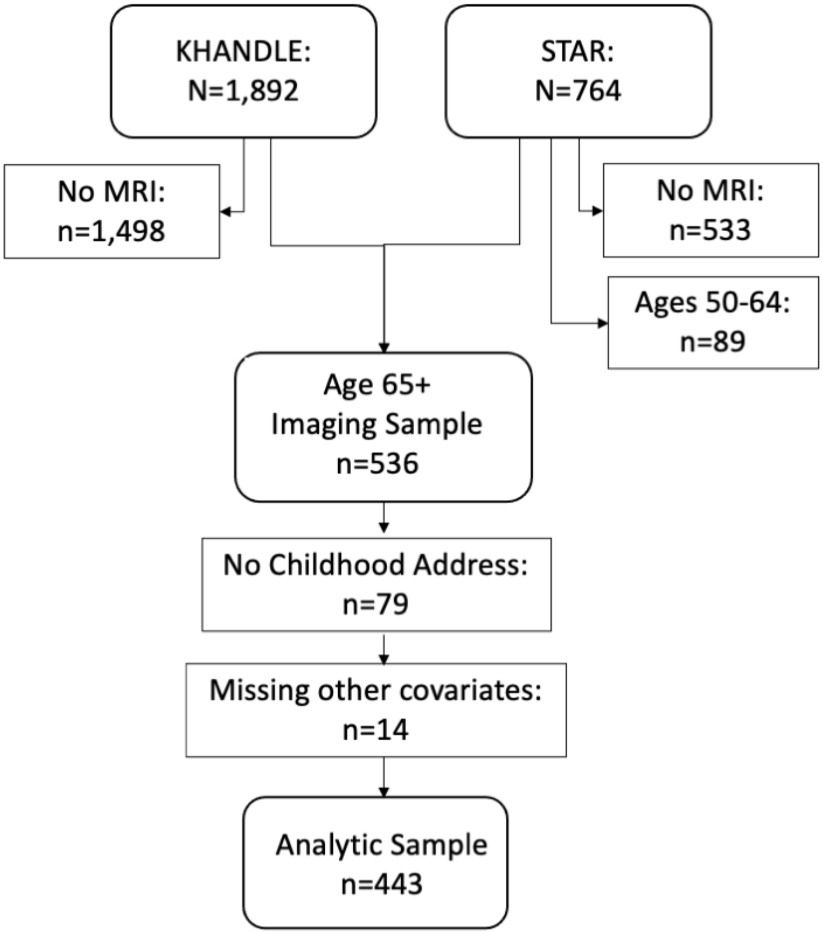

eFigure 2. Flow diagram of the inclusion and exclusion criteria for the analytic sample of the Kaiser Healthy Aging and Diverse Life Experiences (KHANDLE) and Study of Aging in African Americans (STAR) cohorts.

© 2024 Peterson RL et al. *JAMA Network Open.*

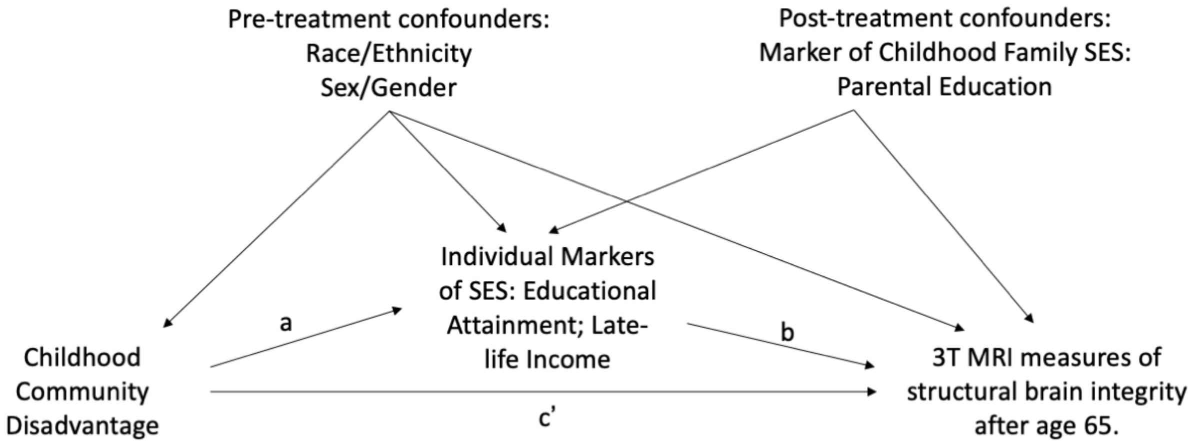

eFigure 3. Directed acyclic graph of tested average direct effects (ADE) of childhood community disadvantage on structural brain volumes and average causal mediated effects (ACME) of participants' education or late-life income on associations of childhood community disadvantage and structural brain volumes, emphasizing key causal mediation assumptions of no unmeasured confounding of the exposure-mediator-outcome (pre-treatment confounding) or mediator-outcome (post-treatment confounding) associations.

© 2024 Peterson RL et al. *JAMA Network Open.*

eAppendix. MRI Methods for the KHANDLE/STAR Cohorts

Removal of Non-brain tissues: The skull is removed using a convolutional neural net method[1] followed by human quality control to provide generally minor cleanup if needed. Structural MRI brain images are then nonlinearly registered performed by a cubic B-spline deformation [2] to a minimal deformation template (MDT) synthetic brain image[3] adapted for age range of 60 and above.

Image Intensity Inhomogeneity Correction: B1 field inhomogeneity is a common problem that limits the precision of image segmentation. We utilize a template-based iterative method for correcting field inhomogeneity bias[4]. At each algorithm iteration, the update of a B-spline deformation between an unbiased template image and the subject image is interleaved with estimation of a bias field based on the current template-to-image alignment. The bias field is modeled using a spatially smooth thin-plate spline interpolation based on ratios of local image patch intensity means between the deformed template and subject images. This is used to iteratively correct subject image intensities which are then used to improve the template-to-image deformation.

Gray, White and CSF Measurement: Our segmentation algorithm is based on an Expectation-Maximization (EM) algorithm that iteratively refines its segmentation estimates to produce outputs that are most consistent with the input intensities from the native-space T1 images along with a model of image smoothness [5, 6]. Like all EM algorithms, the system must be initialized with a reasonable estimate. We produce this initial estimate from the template-space warps of previously segmented images; because locations of WM/GM/CSF tissues are known in the template space, transforming these masks back to each image's native space produces rough estimate 3-tissue segmentations. We then calculate the mean and standard deviation of the image intensities in locations labeled as each tissue type. These values then form the initial parameters for a Gaussian model of image intensity for each class. At each iteration, the algorithm uses a Gaussian model of T1-weighted image intensity for each tissue class, in order to produce a segmentation. In the first iteration, these models are estimated as described above. The segmentation yielded by these appearance models alone is then refined using a Markov Random Field (MRF) model, a computational statistical method that efficiently produces a label map consistent with both the input intensities and image smoothness statistics. Inference in the MRF is computed using an adaptive priors model[6]. This refined segmentation from the MRF is then used to compute new Gaussian intensity models for each tissue class, and the algorithm repeats, iteratively switching between calculating Gaussian appearance models and MRF-based segmentation, until convergence. The MRF-based segmentation at the final iteration is used as the final output segmentation.

White Matter Hyperintensity: WMH is performed on a combination of FLAIR and 3D T1 images using a modified Bayesian probability structure based on a previously published method of histogram fitting [7]. Prior probability maps for WMH were created from more than 700 individuals with semi-automatic detection of WMH followed by manual editing. Likelihood estimates of the native image are calculated through histogram segmentation and thresholding. All segmentation is initially performed in standard space resulting in probability likelihood values of WMH at each voxel in the white matter. These probabilities are then thresholded at 3.5 sd above the mean to create a binary WMH mask. Further segmentation is based on a modified Bayesian approach that combines image likelihood estimates, spatial priors, and tissue class constraints. The segmented WMH masks are then back transformed on to native space for tissue volume calculation. Reliability of this method is well-established[8]

Automatic Hippocampal Segmentation: MRI-derived hippocampal volumetry has been a widely used biomarker in AD to improve early diagnosis[9], enrich subject selection[10], and monitor

© 2024 Peterson RL et al. *JAMA Network Open.*

treatment efficacy[11, 12]. To address this need, the EADC-ADNI Working Group established a Delphi panel to determine the optimum protocol[13], selected orientation parameters[14] and developed the final, rigorously tested protocol along with making publicly available labels from over 100 ADNI subjects[15]. Our hippocampal segmentation method employs a standard atlas based diffeomorphic approach[16] with the minor modification of label refinement. We further modified this approach to include the EADC-ADNI harmonized hippocampal masks to assure standardization across cohorts. Therefore we have adopted the following approach: 1) Subject image pre-processing with extraction of intracranial cavity, non-uniformity correction, tissue classification as discussed above; 2) Atlas Registration of all EADC-ADNI hippocampal masks[9, 13, 15, 17, 18] to each subject; 3) Atlas Fusion utilizing MALF [19, 20]; and 4) Intensity-based label refinement.

ROI-based Analysis: Software developed by the IDeA laboratory allows the creation of any set of user-defined ROIs or utilization of published ROIs. The lab provides multiple sets of predefined regions of interest including lobar volumes, the Desikan-Killiany-Tourville Atlas[21]. Regional measures are calculated by back transformation of the atlas into segmented image native space. A voting scheme is used to assure precise labelling of each region after interpolation of the atlas into native space.

Cortical Thickness: We utilize a registration based method based on Das et al.[22, 23] which consists of the following steps: an initial probabilistic segmentation of GM, WM and CSF after intensity inhomogeneity correction[4] using our segmentation methods [6]. From the three probability maps, a three-label image is formed by picking the tissue type with the highest probability at each voxel. A greedy diffeomorphic registration algorithm is then used to expand the WM segment, to match the GM + WM segment or until a maximum of 6 mm displacement is reached. For each boundary voxel on the GM/WM boundary, the thickness is calculated as the distance moved under the registration transformation, and this thickness value is then propagated across the GM mask.

Infarcts: The presence of MRI infarction was determined from the size, location and imaging characteristics of the lesion. The image analysis system allowed for superimposition of the subtraction image, the proton density image and the T2 weighted image at three times magnified view to assist in interpretation of lesion characteristics. Signal void, best seen on the T2 weighted image was interpreted to indicate a vessel. Only lesions 3mm or larger qualified for consideration as cerebral infarcts. Other necessary imaging characteristics included: 1) CSF density on the subtraction image and 2) If the stroke was in the basal ganglia area, distinct separation from the circle of Willis vessels. Kappa values for agreement amongst the three raters are generally good and range from 0.73 to 0.90.

DTI-derived metrics: The processing steps for all DTI data sets included movement and eddy current correction, gradient direction correction, and brain extraction before fitting the respective models. Eddy current correction and brain extraction was performed using the FSL toolkit[24] (FMRIB software library; http://fsl.fmrib.ox.ac.uk/fsl/fslwiki). Briefly, the FW model considers two co-existing compartments per voxel[25]: one compartment is a free-water compartment, which models isotropic diffusion with a diffusion coefficient of water at body temperature (37 °C) fixed to 3×10^{-3} mm^2/s. The FW fraction is expected to predominantly highlight water molecules in the extracellular space. The second compartment is the tissue compartment, which accounts for all other molecules, i.e., all intra- and extracellular molecules that are hindered or restricted by tissue membranes[26]. The FW-DTI model is described by:

$$S_i = S_0 \left[(1-f)\exp\left(-b_i g_i^T D g_i\right) + f exp\left(-bD_{iso}\right)\right] \qquad \textit{Equation 1}$$

where S_i and S_0 are the signal from the i-th diffusion and non-diffusion weighted measurements, respectively, $D_{iso} = 3\times10^{-3}mm^2/sec$ is the isotropic free water diffusivity, D is the tissue diffusion tensor, b_i and g_i are the diffusion-weighting amplitude (in mm^2/s) and unit gradient encoding vector, respectively. Single-shell (i=1) and multi-shell data were processed with algorithms that have been previously described[27, 28].

The methods contains the following steps: 1) the tissue compartment is modeled by a diffusion tensor characterizing the "tissue" molecules, as well as the fractional volume of the free-water compartment in each voxel, resulting in the FW and FA maps (see Equation 1), 2) the individual FA map is linearly and non-linearly registered to the standard FSL FA template space (FMRIB 1-mm FA template) using linear and nonlinear transformations, 3) the resulting transformation parameters are applied to the FW and FA maps, 4) a WM mask is defined by thresholding the FSL FA template at a value of 0.3 to reduce cerebrospinal fluid (CSF) partial volume contamination[29], 5) overall measures of mean FW and mean FA are computed by superimposing the WM mask onto the individual co-registered FW and FA maps and averaging values within these WM voxels.

References for MRI Methods

1. Fletcher E, DeCarli C, Fan AP, Knaack A. Convolutional Neural Net Learning Can Achieve Production-Level Brain Segmentation in Structural Magnetic Resonance Imaging. Front Neurosci 2021;15:683426.
2. Rueckert D, Aljabar P, Heckemann RA, Hajnal JV, Hammers A. Diffeomorphic registration using B-splines. Med Image Comput Comput Assist Interv 2006;9:702-709.
3. Kochunov P, Lancaster JL, Thompson P, et al. Regional spatial normalization: toward an optimal target. J Comput Assist Tomogr 2001;25:805-816.
4. Fletcher E, Carmichael O, Decarli C. MRI non-uniformity correction through interleaved bias estimation and B-spline deformation with a template. Conf Proc IEEE Eng Med Biol Soc 2012;2012:106-109.
5. Rajapakse JC, Giedd JN, DeCarli C, et al. A technique for single-channel MR brain tissue segmentation: application to a pediatric sample. Magnetic Resonance Imaging 1996;14:1053-1065.
6. Fletcher E, Singh B, Harvey D, Carmichael O, Decarli C. Adaptive image segmentation for robust measurement of longitudinal brain tissue change. Conf Proc IEEE Eng Med Biol Soc 2012;2012:5319-5322.
7. DeCarli C, Miller BL, Swan GE, et al. Predictors of brain morphology for the men of the NHLBI twin study. Stroke 1999;30:529-536.
8. Maillard P, Lu H, Arfanakis K, et al. Instrumental validation of free water, peak-width of skeletonized mean diffusivity, and white matter hyperintensities: MarkVCID neuroimaging kits. Alzheimers Dement (Amst) 2022;14:e12261.
9. Frisoni GB, Bocchetta M, Chetelat G, et al. Imaging markers for Alzheimer disease: which vs how. Neurology 2013;81:487-500.
10. Lorenzi M, Donohue M, Paternico D, et al. Enrichment through biomarkers in clinical trials of Alzheimer's drugs in patients with mild cognitive impairment. Neurobiol Aging 2010;31:1443-1451, 1451 e1441.
11. Hampel H, Frank R, Broich K, et al. Biomarkers for Alzheimer's disease: academic, industry and regulatory perspectives. Nat Rev Drug Discov 2010;9:560-574.
12. Hampel H, Wilcock G, Andrieu S, et al. Biomarkers for Alzheimer's disease therapeutic trials. Prog Neurobiol 2011;95:579-593.

© 2024 Peterson RL et al. *JAMA Network Open.*

13. Boccardi M, Bocchetta M, Apostolova LG, et al. Delphi definition of the EADC-ADNI Harmonized Protocol for hippocampal segmentation on magnetic resonance. Alzheimers Dement 2014.
14. Boccardi M, Bocchetta M, Apostolova LG, et al. Establishing magnetic resonance images orientation for the EADC-ADNI manual hippocampal segmentation protocol. J Neuroimaging 2014;24:509-514.
15. Bocchetta M, Boccardi M, Ganzola R, et al. Harmonized benchmark labels of the hippocampus on magnetic resonance: The EADC-ADNI project. Alzheimers Dement 2014.
16. Vercauteren T, Pennec X, Perchant A, Ayache N. Non-parametric diffeomorphic image registration with the demons algorithm. Med Image Comput Comput Assist Interv 2007;10:319-326.
17. Boccardi M, Bocchetta M, Ganzola R, et al. Operationalizing protocol differences for EADC-ADNI manual hippocampal segmentation. Alzheimers Dement 2013.
18. Frisoni GB, Jack CR. HarP: The EADC-ADNI Harmonized Protocol for manual hippocampal segmentation. A standard of reference from a global working group. Alzheimers Dement 2015;11:107-110.
19. Wang H, Suh JW, Das SR, Pluta J, Craige C, Yushkevich PA. Multi-Atlas Segmentation with Joint Label Fusion. IEEE Trans Pattern Anal Mach Intell 2012.
20. Wang H, Yushkevich PA. Dependency Prior for Multi-Atlas Label Fusion. Proc IEEE Int Symp Biomed Imaging 2012;2012:892-895.
21. Klein A, Tourville J. 101 labeled brain images and a consistent human cortical labeling protocol. Front Neurosci 2012;6:171.
22. Das SR, Avants BB, Grossman M, Gee JC. Registration based cortical thickness measurement. Neuroimage 2009;45:867-879.
23. Tustison NJ, Cook PA, Klein A, et al. Large-scale evaluation of ANTs and FreeSurfer cortical thickness measurements. NeuroImage 2014;99:166-179.
24. Jenkinson M, Beckmann CF, Behrens TE, Woolrich MW, Smith SM. Fsl. NeuroImage 2012;62:782-790.
25. Pierpaoli C, Basser PJ. Toward a quantitative assessment of diffusion anisotropy. Magn Reson Med 1996;36:893-906.
26. Pasternak O, Sochen N, Gur Y, Intrator N, Assaf Y. Free water elimination and mapping from diffusion MRI. Magn Reson Med 2009;62:717-730.
27. Maillard P, Lu H, Arfanakis K, et al. Instrumental validation of free water, peak-width of skeletonized mean diffusivity, and white matter hyperintensities: MarkVCID neuroimaging kits. Alzheimer's & Dementia: Diagnosis, Assessment & Disease Monitoring 2022;14:e12261.
28. Hoy AR, Koay CG, Kecskemeti SR, Alexander AL. Optimization of a free water elimination two-compartment model for diffusion tensor imaging. NeuroImage 2014;103:323-333.
29. Smith SM, Kindlmann G, Jbabdi S. Chapter 10 - Cross-Subject Comparison of Local Diffusion MRI Parameters. In: Johansen-Berg H, Behrens TEJ, eds. Diffusion MRI (Second Edition). San Diego: Academic Press, 2014: 209-239.

© 2024 Peterson RL et al. *JAMA Network Open.*
