# Supplementary material for: Childhood Community Disadvantage and MRI-Derived Structural Brain Integrity After Age 65 Years
Source: JAMA Netw Open. 2024 Nov 7;7(11):e2443703. doi: 10.1001/jamanetworkopen.2024.43703 (PMC11544493; doi:10.1001/jamanetworkopen.2024.43703)
Supplement: Supplement 2. — Data Sharing Statement [file jamanetwopen-e2443703-s002.pdf]

Data Sharing Statement

Peterson. Childhood Community Disadvantage and MRI-Derived Structural Brain Integrity After Age 65 Years. *JAMA Netw Open*. Published November 07, 2024.
doi:10.1001/jamanetworkopen.2024.43703

Data
Data available: No

Additional Information
Explanation for why data not available: Requests to use data from the KHANDLE and STAR cohorts can be submitted to the MPIs at https://sites.google.com/g.ucla.edu/khandle-study-site/project-proposals-new-data-user-registration?authuser=0
